# Supplementary material for: PlantaNet and PlantaNetLite: Efficient and explainable multi-crop plant disease classification via transformer benchmarking and custom lightweight CNNs
Source: PLoS One. 2026 Apr 24;21(4):e0346977. doi: 10.1371/journal.pone.0346977 (PMC13108818; doi:10.1371/journal.pone.0346977)
Supplement: Attachment — Submitted filename: The dataset description is ambiguous because the m.docx [file pone.0346977.s001.docx]

1. The dataset description is ambiguous because the manuscript states that images were aggregated from multiple public repositories with manual verification, while the Data Availability section specifies a single Kaggle dataset, making it unclear which exact data version was used for experiments.
2. The augmentation factor is conceptually unclear, as the paper states that each original image is augmented three times, but the final dataset size (136,482 from ~45,000 images) does not clearly indicate whether the original image is retained alongside three augmented copies or replaced by them.

3. The stratified split procedure lacks sufficient detail, as the manuscript does not explicitly state the split ratios or whether augmentation was applied strictly after splitting, which affects reproducibility.

4. Training configuration details are incomplete, as the manuscript does not specify the learning rate scheduler, warm-up strategy, label smoothing coefficient, or dropout probabilities, all of which can materially affect model performance.

5. The optimization setup for transformer baselines is insufficiently described, particularly regarding whether pretrained weights were used and whether the same optimizer and regularization strategy were applied as for the CNN models.

6. The loss and accuracy values reported in Table 5 are internally inconsistent, most notably for XCiT-Tiny and PlantaNet, where near-perfect accuracies are paired with unusually high cross-entropy losses, suggesting either reporting errors or inconsistent loss definitions.

7. The text claims that CAFormer-s18 exhibits extremely low losses, but this statement conflicts with the high-loss values reported for other models in the same table without explanation of differing loss formulations.

8. The manuscript states that PlantaNet achieves the best overall performance among all models, yet the transformer baselines are not evaluated on the test set, making this claim unsupported by a unified comparison.

9. Hyperparameter tuning results in Table 8 report best validation accuracy values around 95–96%, which directly contradicts the 99.5% validation accuracy reported elsewhere, without clarification that tuning was performed under a reduced epoch budget.

10. The discussion of hyperparameter tuning does not explicitly explain that configurations were selected after 10 epochs and then retrained for 100 epochs, which creates confusion about which results correspond to final models.

11. The term “BFIT tiny” appears in the results discussion, but no such model is defined elsewhere, and this appears to be a typographical error referring to “BiT-Tiny”.

12. Model naming is inconsistent across the manuscript, with variations such as “CAFormer s18” versus “CAFormer-s18” and “PVT 2” versus “PVT-v2”, which reduces clarity.

13. Section numbering beginning with 0.x throughout the manuscript is non-standard for journal articles and may indicate a formatting or template issue.

14. Several tables contain broken words and hyphenation artifacts (e.g., “transformer”, “Albumentations”), which appear to be PDF rendering errors and should be corrected before submission.

15. The reference list contains multiple duplicate entries explicitly labeled as “Duplicate of refXX”, which is inappropriate for a final journal submission and should be consolidated.

16. The explainability analysis relies on a single correctly classified sample per class, which is insufficient to support generalized claims about model interpretability and may be vulnerable to selection bias.

17. No quantitative evaluation of explainability quality is provided, such as overlap with lesion regions or comparison on misclassified samples, limiting the strength of the interpretability claims.

18. The latency and inference-time measurements lack sufficient context, as the manuscript does not specify batch size, hardware model, or averaging protocol, which are necessary for fair efficiency comparisons.

19. Although real-world deployment is emphasized, the dataset largely originates from curated or semi-controlled sources, and no external or field dataset validation is performed to substantiate real-world robustness claims.

20. The absence of ablation studies makes it difficult to assess the individual contribution of design choices such as GroupNorm, MixUp, label smoothing, and input resolution to the reported performance gains.
