# Supplementary material for: PlantaNet and PlantaNetLite: Efficient and explainable multi-crop plant disease classification via transformer benchmarking and custom lightweight CNNs
Source: PLoS One. 2026 Apr 24;21(4):e0346977. doi: 10.1371/journal.pone.0346977 (PMC13108818; doi:10.1371/journal.pone.0346977)
Supplement: Attachment — Submitted filename: Response to Reviewers.docx [file pone.0346977.s002.docx]

**1.** The dataset description is ambiguous because the manuscript states that images were aggregated from multiple public repositories with manual verification, while the Data Availability section specifies a single Kaggle dataset, making it unclear which exact data version was used for experiments

**Response to Comment 1:** We agree that the dataset description in the original manuscript was not sufficiently clear and could create confusion regarding the exact data source used in our experiments.

In this study, the dataset construction was guided by the objective of targeting plant species that are agriculturally significant in Bangladesh. To build a comprehensive dataset covering these crops, we collected non-augmented images from multiple publicly available Kaggle repositories. The datasets used include:

- [PlantVillage dataset](https://www.kaggle.com/datasets/mohitsingh1804/plantvillage) (Apple, Blueberry, Corn, Grape, Orange, Soybean, Strawberry)
- [Plant Disease dataset](https://www.kaggle.com/datasets/emmarex/plantdisease) (Pepper bell, Tomato, Potato)
- [Mango Leaf Diseases](https://www.kaggle.com/datasets/ahmadzargar/mango-leaf-diseases-dataset) dataset (Mango)
- [Rice Leaf Disease](https://www.kaggle.com/datasets/dedeikhsandwisaputra/rice-leafs-disease-dataset) dataset (Rice)
- [Banana Leaf Disease](https://www.kaggle.com/datasets/shifatearman/bananalsd) dataset (Banana)
- [Bean Disease dataset](https://www.kaggle.com/datasets/therealoise/bean-disease-dataset) (Bean)

Each dataset was downloaded in its original form (without prior augmentation), manually verified, cleaned when necessary, and then aggregated into a unified dataset. The final combined dataset was used for all training, validation, and testing experiments described in the manuscript.

The ambiguity arose because the Data Availability section mentioned a single Kaggle source, whereas the dataset was in fact constructed by integrating multiple publicly available Kaggle datasets. We have now revised the Dataset Description and Data Availability sections to explicitly list all source repositories and clarify that the experimental dataset is an aggregated and manually verified compilation of these sources.

These revisions ensure full transparency regarding the exact data version used in our experiments and improve reproducibility.

**2.** The augmentation factor is conceptually unclear, as the paper states that each original image is augmented three times, but the final dataset size (136,482 from ~45,000 images) does not clearly indicate whether the original image is retained alongside three augmented copies or replaced by them.

**Response to Comment 2:** We agree that the description of the augmentation factor in the original manuscript did not clearly specify whether the original images were retained or replaced, which may have led to confusion regarding the final dataset size.

The augmentation procedure was implemented using the Albumentations library in combination with OpenCV. The dataset contained 51 class-specific subfolders, and augmentation was applied independently within each class to maintain label consistency. Only images with extensions .png, .jpg, and .jpeg were processed.

For each image, the following preprocessing and augmentation pipeline was applied:

1. The image was read using OpenCV and converted from BGR to RGB format.
2. A composed transformation pipeline with probabilistic geometric and photometric operations was applied, including:
   - HorizontalFlip (p = 0.5)
   - VerticalFlip (p = 0.5)
   - RandomRotate90 (p = 0.5)
   - Affine transformation (p = 0.5) with scaling [0.9, 1.1], translation up to ±5%, rotation [−25°, 25°], shear [−10°, 10°], and bilinear interpolation
   - RandomBrightnessContrast (p = 0.5)
   - Resize to 224 × 224 pixels (applied deterministically)

All probabilistic operations were independently sampled during each pipeline execution, ensuring that repeated passes of the same image produced different stochastic outputs.

The augmentation was applied as follows:

- First, each original image was processed and resized to 224 × 224 pixels. This resized version was retained as part of the dataset (ensuring uniform spatial dimensions across all samples).
- Then, the same original image was passed through the identical augmentation pipeline two additional times.
- These two augmented variants were saved as new files using distinct filenames (e.g., originalname_aug1, originalname_aug2).

Therefore, for each original image N, the final dataset contains:

- One resized base image (retained sample)
- Two additional independently augmented variants

This results in a final dataset size of approximately 3N. The original samples were not discarded; rather, they were preserved (after resizing) alongside two newly generated augmented copies.

We have revised the manuscript to explicitly clarify this process and to state that the reported final dataset size (136,482 images) reflects retention of the resized original images plus two augmented variants per sample. This revision removes the ambiguity regarding the effective 3× augmentation factor and ensures transparency in dataset construction.

**3.** The stratified split procedure lacks sufficient detail, as the manuscript does not explicitly state the split ratios or whether augmentation was applied strictly after splitting, which affects reproducibility.

**Response to Comment 3:** We agree that the original manuscript did not provide sufficient detail regarding the stratified splitting procedure and the order of splitting and augmentation, which is essential for full reproducibility.

The dataset was partitioned using a class-wise stratified splitting strategy, where each class directory was processed independently to preserve the original class distribution across all subsets. The dataset was divided into **70% training, 15% validation, and 15% testing**, and the splitting was implemented using the train_test_split function from scikit-learn with a fixed random_state = 42 to ensure deterministic and reproducible results.

The splitting process was performed in two stages for each class:

1. First, 70% of the images were assigned to the training set, while the remaining 30% were temporarily grouped.
2. The remaining 30% were then evenly divided into validation and test subsets, resulting in the final 70:15:15 split.

Importantly, data augmentation was applied **strictly after** the dataset splitting and **only to the training subset**. The validation and test sets were not augmented, except for deterministic resizing to the required input resolution. This protocol prevents data leakage and ensures that augmented variants of the same original image do not appear across different subsets.

Formally, for an original dataset of N images per class, the partitioning results in approximately 0.7N training images, 0.15N validation images, and 0.15N test images. Augmentation was then applied exclusively to the training set according to the defined augmentation factor, while the validation and test sets remained fixed for unbiased evaluation.

We have revised the manuscript to explicitly state the split ratios, random seed, class-wise stratification strategy, and the chronological order of splitting and augmentation. These additions ensure that the experimental protocol is fully transparent and reproducible.

**4.** Training configuration details are incomplete, as the manuscript does not specify the learning rate scheduler, warm-up strategy, label smoothing coefficient, or dropout probabilities, all of which can materially affect model performance.

**Response to Comment 4:** We agree that explicitly specifying the learning rate scheduler, warm-up strategy, label smoothing, and dropout parameters is essential for reproducibility and accurate performance interpretation.

In our study, a unified training configuration was applied consistently across the majority of the evaluated models to ensure fair comparison. All models were initialized using ImageNet-pretrained weights obtained through the timm library when available. Training was conducted for a maximum of 50 epochs using the AdamW optimizer with an initial learning rate of 3 * 10^-5^ and weight decay of 1*10^-4^

A cosine annealing learning rate scheduler was employed with (T_max_ = 50) epochs. No learning rate warm-up strategy was used. Cross-entropy loss without label smoothing (epsilon = 0) was applied uniformly. The default dropout configurations provided in the respective model implementations were retained, and no additional dropout layers or modified dropout probabilities were introduced.

The batch size was set to 128. Training was performed using automatic mixed precision (AMP) to improve computational efficiency. Early stopping with a patience of 5 epochs was applied based on validation accuracy, and the best-performing model checkpoint was selected for final evaluation.

All input images were resized to (224 * 224) pixels and normalized using ImageNet mean and standard deviation values.

We have revised the manuscript to explicitly include these training configuration details and to clarify that this setup was applied consistently across the evaluated models. These additions ensure full transparency and reproducibility of the experimental protocol.

**5.** The optimization setup for transformer baselines is insufficiently described, particularly regarding whether pretrained weights were used and whether the same optimizer and regularization strategy were applied as for the CNN models.

**Response to Comment 5:** We agree that the training protocol for the transformer baselines was not described with sufficient clarity in the original submission.

In our experiments, all transformer baseline models (CAFormer-s18, BiT-Tiny, RepViT, PiT, PVT-v2, ViT-Tiny, XCiT-Tiny, and DeiT-Tiny) were initialized using ImageNet-1k pretrained weights obtained through the official timm implementations. For each model, the final classification layer was replaced with a newly initialized fully connected layer corresponding to the 51 target classes, and the entire network was fine-tuned on the plant disease dataset.

To ensure consistency across experiments, we used the same optimizer (Adamax), initial learning rate (1 × 10⁻³), weight decay (1 × 10⁻⁴), batch size (32), and input resolution (160 × 160) for both transformer baselines and the proposed CNN models. The same normalization and preprocessing pipeline was applied in all cases.

We note that MixUp regularization was used only during CNN training, as part of the architectural optimization process for PlantaNet and PlantaNetLite. The transformer baselines were fine-tuned following a standard pretrained fine-tuning setup without additional data mixing strategies. Label smoothing (coefficient = 0.1) was applied consistently across models.

These details have now been added to the revised manuscript in the Baseline Benchmarking section to make the experimental setup fully transparent and reproducible.

**6.** The loss and accuracy values reported in Table 5 are internally inconsistent, most notably for XCiT-Tiny and PlantaNet, where near-perfect accuracies are paired with unusually high cross-entropy losses, suggesting either reporting errors or inconsistent loss definitions.

**Response to Comment 6:** We thank the reviewer for identifying this issue. Upon re-examination, we found that the originally reported loss values for XCiT-Tiny and PlantaNet were not computed consistently with the other models. Specifically, the values reflected an intermediate logging output rather than the final epoch cross-entropy loss used for performance comparison.

We have re-trained and re-evaluated these models using the same experimental protocol applied to all baselines. All reported training and validation losses in Table 5 now correspond to the final epoch cross-entropy loss, computed using the same loss function and reporting procedure across models.

The updated results are internally consistent, with near-perfect accuracies now accompanied by appropriately low cross-entropy values. Table 5 has been corrected accordingly in the revised manuscript.

**7.** The text claims that CAFormer-s18 exhibits extremely low losses, but this statement conflicts with the high-loss values reported for other models in the same table without explanation of differing loss formulations.

**Response to Comment 7:** The concern arose because the originally reported loss values for two models (XCiT-Tiny and PlantaNet) were inconsistent with the evaluation protocol used for the rest of the models, which made CAFormer-s18 appear disproportionately low in comparison. After correcting those rows using the same final-epoch evaluation procedure, the losses across models are now internally consistent and directly comparable.

We have also revised the wording in the Results section to avoid overstating differences in loss magnitude. Instead of describing CAFormer-s18 as exhibiting “extremely low” loss, we now state that it achieved strong validation performance with stable convergence under the same evaluation protocol.

These revisions remove the apparent contradiction and ensure clarity in cross-model comparison.

**8.** The manuscript states that PlantaNet achieves the best overall performance among all models, yet the transformer baselines are not evaluated on the test set, making this claim unsupported by a unified comparison.

**Response to Comment 8:** We acknowledge that in the original manuscript, test set evaluation was reported only for the proposed CNN models, while transformer baselines were compared primarily using validation performance. Therefore, the statement that PlantaNet achieved the “best overall performance” was overly strong.

We have revised the manuscript to remove this claim and instead emphasize that PlantaNet achieved competitive validation performance relative to transformer baselines while offering a favorable accuracy–efficiency trade-off. The conclusions have been updated accordingly to ensure that all claims are fully supported by the presented results.

**9.** Hyperparameter tuning results in Table 8 report best validation accuracy values around 95–96%, which directly contradicts the 99.5% validation accuracy reported elsewhere, without clarification that tuning was performed under a reduced epoch budget.

**Response to Comment 9:** The validation accuracies reported in Table 8 correspond to the hyperparameter search phase, during which each configuration was trained for only 10 epochs to enable efficient comparison. These early-stage validation accuracies (approximately 95–96%) reflect partial convergence and were used solely to select the most promising configuration.

After selecting the best-performing configuration (Config-3), the model was retrained from scratch for 100 epochs under the full training schedule. The final validation accuracy of approximately 99.5% reported elsewhere in the manuscript corresponds to this complete 100-epoch training.

We have revised the manuscript to explicitly clarify this distinction and avoid confusion between the hyperparameter search phase and the final model training results.

**10.** The discussion of hyperparameter tuning does not explicitly explain that configurations were selected after 10 epochs and then retrained for 100 epochs, which creates confusion about which results correspond to final models.

**Response to Comment 10:** We appreciate the reviewer’s observation. In the original manuscript, the distinction between the hyperparameter search phase and the final full training phase was not described clearly enough.

Hyperparameter tuning was conducted using a reduced training budget of 10 epochs per configuration to enable efficient comparison across multiple parameter combinations. The configuration achieving the best early validation performance (Config-3) was then selected and retrained from scratch for the full 100-epoch schedule.

All final performance metrics reported for PlantaNet and PlantaNetLite (including the 99.5% validation accuracy) correspond to this complete 100-epoch training, not to the intermediate tuning phase.

We have revised the manuscript to explicitly describe this two-stage training procedure to avoid confusion.

**11.** The term “BFIT tiny” appears in the results discussion, but no such model is defined elsewhere, and this appears to be a typographical error referring to “BiT-Tiny”.

**Response to Comment 11:** The term “BFIT tiny” in the Results section was unintended and should read “BiT-Tiny.” We have corrected this throughout the manuscript and ensured consistent naming of all baseline models at first mention, including appropriate citations.

**12.** Model naming is inconsistent across the manuscript, with variations such as “CAFormer s18” versus “CAFormer-s18” and “PVT 2” versus “PVT-v2”, which reduces clarity.

**Response to Comment 12:** Thank you for highlighting the inconsistency in model naming. We have carefully reviewed the manuscript and standardized the naming conventions of all baseline models to ensure clarity and consistency. For example, we now consistently use “CAFormer-s18” and “PVT-v2” throughout the manuscript. Additionally, we verified that each model is introduced with its full name and appropriate citation at first mention. These corrections improve readability and presentation consistency.

**13.** Section numbering beginning with 0.x throughout the manuscript is non-standard for journal articles and may indicate a formatting or template issue.

**Response to Comment 13:** The section numbering beginning with “0.x” was unintended and resulted from template configuration during manuscript preparation. We have corrected the section numbering throughout the manuscript to follow standard journal formatting conventions, beginning from Section 1. These changes improve the clarity and presentation of the manuscript.

**14.** Several tables contain broken words and hyphenation artifacts (e.g., “transformer”, “Albumentations”), which appear to be PDF rendering errors and should be corrected before submission.

**Response to Comment 14:** The broken words and hyphenation artifacts in several tables were unintended formatting issues resulting from PDF rendering and table layout settings. We have corrected these artifacts, removed automatic hyphenation, and carefully reviewed all tables to ensure proper word formatting and clarity. The revised manuscript no longer contains these formatting inconsistencies.

**15.**  The reference list contains multiple duplicate entries explicitly labeled as “Duplicate of refXX”, which is inappropriate for a final journal submission and should be consolidated.

**Response to Comment 15:** The duplicate reference entries labeled as “Duplicate of refXX” were unintended artifacts introduced during manuscript revision and reference management. We have carefully reviewed the reference list, removed all duplicate entries, consolidated repeated citations, and verified that in-text citations correspond correctly to the updated reference numbering. The reference list has been fully cleaned and standardized according to journal formatting guidelines.

**16.** The explainability analysis relies on a single correctly classified sample per class, which is insufficient to support generalized claims about model interpretability and may be vulnerable to selection bias.

**Response to Comment 16:** The Grad-CAM and Grad-CAM++ visualizations were included to provide qualitative insight into the model’s attention patterns rather than to serve as a comprehensive interpretability evaluation. We agree that a single example per class does not constitute a statistically exhaustive analysis. In the revised manuscript, we have clarified that the presented samples are illustrative and have revised the language to avoid overgeneralized claims. We now explicitly state that a broader quantitative assessment of interpretability is beyond the scope of the current study and represents an important direction for future work.

**17.** No quantitative evaluation of explainability quality is provided, such as overlap with lesion regions or comparison on misclassified samples, limiting the strength of the interpretability claims.

**Response to Comment 17:** We appreciate the reviewer’s suggestion regarding quantitative evaluation of explainability quality. The Grad-CAM and Grad-CAM++ analyses in our study were intended to provide qualitative insight into model attention patterns rather than to serve as a formal localization benchmark. The dataset used in this work does not provide pixel-level lesion annotations, which limits the ability to compute overlap-based metrics such as localization IoU.

In the revised manuscript, we have clarified that the explainability analysis is qualitative and have revised the text to avoid overstating interpretability claims. We also explicitly acknowledge that a quantitative evaluation of localization performance would further strengthen interpretability assessment and represents an important direction for future research.

**18.** The latency and inference-time measurements lack sufficient context, as the manuscript does not specify batch size, hardware model, or averaging protocol, which are necessary for fair efficiency comparisons.

**Response to Comment 18:** We agree that latency measurements require clear reporting of experimental conditions. In the revised manuscript, we have added a detailed description of the inference protocol, including the hardware environment (NVIDIA Tesla T4 GPU on Kaggle), batch size (1), evaluation mode settings, and averaging procedure (30 forward passes with CUDA synchronization). These clarifications ensure that the reported latency values are transparent and reproducible.

**19.** Although real-world deployment is emphasized, the dataset largely originates from curated or semi-controlled sources, and no external or field dataset validation is performed to substantiate real-world robustness claims.

**Response to Comment 19:** While our dataset aggregates images from multiple publicly available repositories and includes diverse crop–disease combinations, we acknowledge that it remains curated and may not fully represent the variability encountered in uncontrolled field environments.

In the revised manuscript, we have moderated claims regarding real-world robustness and clarified that our conclusions are limited to the dataset used in this study. We also explicitly state that additional validation on independently collected field datasets would further strengthen claims regarding practical deployment. The emphasis on lightweight design and latency reflects computational suitability for deployment rather than validated field robustness.

**20.** The absence of ablation studies makes it difficult to assess the individual contribution of design choices such as GroupNorm, MixUp, label smoothing, and input resolution to the reported performance gains.

**Response to Comment 20:** We thank the reviewer for this constructive suggestion. To better clarify the role of the proposed architectural choices, we performed additional ablation experiments on PlantaNet by modifying one component at a time while keeping the remaining training settings unchanged. Specifically, we examined three architectural elements: (i) replacing Group Normalization with Batch Normalization, (ii) removing dropout regularization, and (iii) removing the additional convolutional refinement block.

The results are presented in the newly added Ablation Study subsection (Section 6.5, Table 9). Replacing Group Normalization with Batch Normalization reduced validation accuracy from 99.37% to 98.90%, suggesting that GroupNorm provides more stable optimization under the selected batch size. Removing dropout led to a slight decrease in validation accuracy (99.37% to 99.11%), indicating its regularization benefit. Excluding the additional convolutional refinement block resulted in a minor reduction (99.37% to 99.28%), implying that while the block contributes to feature refinement, its impact is incremental rather than dominant.

During this analysis, training-related settings such as MixUp and label smoothing were kept fixed in order to isolate the effect of architectural changes. These results help clarify the contribution of the main structural components and strengthen the empirical support for the final model design.
